# Perceived Driving Difficulty, Negative Affect, and Emotion Dysregulation in Self-Identified Autistic Emerging Drivers

**DOI:** 10.3389/fpsyg.2022.754776

**Published:** 2022-01-31

**Authors:** Megan Fok, Justin M. Owens, Thomas H. Ollendick, Angela Scarpa

**Affiliations:** ^1^Virginia Tech Autism Clinic & Center for Autism Research, Blacksburg, VA, United States; ^2^Department of Psychology, Virginia Tech, Blacksburg, VA, United States; ^3^Division of Vehicle, Driver & Safety Systems, Virginia Tech Transportation Institute, Blacksburg, VA, United States

**Keywords:** adults, driving, emotion regulation, negative affect, transition planning

## Abstract

Driving is central to adult independence and autonomy; yet most autistic young adults do not acquire driver’s licenses. It is important to understand barriers to achieving this milestone for autistic adults. Differences in negative affect and emotion dysregulation associated with autism may interfere with managing difficult driving situations. The current study compared perceived driving difficulty (DD), emotion dysregulation, and negative affect in emerging drivers with and without autistic traits (AT), and investigated how emotion dysregulation and negative affect relate to perceived DD. We expected (1) greater perceived DD, emotion dysregulation, and negative affect in participants with AT and (2) a positive correlation of perceived DD with both emotion dysregulation and negative affect in the whole sample. Thirty-seven adolescents and young adults (15 AT) self-reported perceived DD in 15 scenarios and completed the Difficulty in Emotion Dysregulation Scale (DERS) and the Depression, Anxiety, and Stress Scale (DASS). Autistic participants scored significantly higher on mean perceived DD, DERS Impulse subscale, DASS total and DASS Stress subscale scores. Perceived DD positively correlated with the DERS and DASS total scores, all DASS subscales, and DERS Nonacceptance, Goals, and Impulse subscales across the whole sample. The findings highlight the roles of emotion dysregulation and negative affect in perceived DD in emerging drivers with AT. In particular, emotional stress and impulsivity may map onto mechanisms of over-reactivity to negative affect and explain why autistic people perceive particular situations as difficult when driving. Implications and directions for future research are discussed.

## Introduction

The Interagency Autism Coordinating Committee (2017) outlined strategic research targets for the transition to adult services and continued lifespan support. Driving can be considered an appropriate transition target, as it is a central skill that enables successful, independent adult living by providing opportunities to increase economic, educational, and social well-being. Driving, for example, has been shown to increase employment, educational, and social opportunities for autistic adults (Feeley, 2010; Daly et al., 2014; Interagency Autism Coordinating Committee, 2017; Curry et al., 2018; Myers et al., 2019). Autistic adolescents are more likely to be enrolled in post-secondary education or employed if they have their driver’s license and are more likely to obtain licensure if their individualized education plans include driving-related goals (Huang et al., 2012). Longitudinal research on outcomes of autistic adults found that increased community and employment opportunities contributed to greater physical and mental health and reduced maladaptive behaviors (Taylor and Mailick, 2014). Altogether, vocational activities are supported by obtaining a driver’s license and can help autistic individuals achieve better overall outcomes.

The primary characteristics of autism^1^ are social communication/interaction differences and repetitive, restricted behaviors (American Psychiatric Association [APA], 2013). These characteristics may not directly hinder skills necessary for safe vehicle control, yet autistic individuals obtain driver’s licenses less frequently, experience greater difficulty learning to drive, and exhibit more maladaptive driving behaviors compared to non-autistic (NA) people (Feeley, 2010; Sheppard et al., 2010; Cox et al., 2012; Reimer et al., 2013; Daly et al., 2014; Curry et al., 2018; Myers et al., 2019). Only 34% of autistic adolescents have their driver’s licenses compared to 83.5% of NA peers, and autistic adolescents acquire their licenses on average nine months later than their peers (Curry et al., 2018). Klin et al. (2003) posited that autistic individuals have poor generalization abilities, preventing the translation of knowledge learned in a structured environment (e.g., explicit rules) to performance in a naturalistic environment, such as driving. Indeed, previous research has used virtual reality to simulate more realistic application of driving skills to autistic youth in a controllable setting with unique, adaptive responsive technology (Bian et al., 2015, 2016; Wade et al., 2016). As such, autistic individuals may benefit from driving instruction that considers their unique learning styles.

Autistic adolescents reportedly struggle with learning the necessary higher-order skills for driving (e.g., multitasking, interpreting other drivers’ non-verbal cues, adapting to unexpected changes) and can benefit from greater parental engagement when learning to drive, individualized driving instruction, and having developed independent daily living skills before beginning to drive (Cox et al., 2012; Myers et al., 2019). While watching videos of other driving scenarios, autistic adults exhibited slower reaction times and identified fewer social hazards, such as pedestrians stepping in front of the vehicle and cyclists crossing the road (Sheppard et al., 2010). Research on the physiological experiences of autistic drivers found increased heart rate and higher eye gaze suggesting greater anxious arousal and less orientation to objects in the driver’s low visual field (e.g., dashboard, directly oncoming vehicles; Reimer et al., 2013). In a simulated driving performance, autistic characteristics, such as insistence on sameness, can affect driving with road detours or unpredictable pedestrian activity and may be correlated with impaired emotional responding (Cox et al., 2020). In addition, autistic drivers experience more difficulties with real driving situations, including driving in traffic, at night, and on highway, and receive more moving violations (Daly et al., 2014; Chee et al., 2017).

Emotion dysregulation can be defined as the inability to modulate affective states and/or to flexibly use strategies to achieve goals and maintain situationally appropriate behaviors (Thompson, 1994). On the Difficulties in Emotion Regulation Scale (DERS; Gratz and Roemer, 2004), in a NA sample, Trógolo et al. (2014) found that the subscales *Nonacceptance*, *Goals*, and *Awareness* predicted poor driving behaviors (e.g., misjudging speed of oncoming car, poor route planning, failing to see pedestrians/other objects), the subscales *Nonacceptance* and *Awareness* predicted anxious driving (e.g., feeling nervous or impatient while driving), and the subscales *Strategies* and *Impulse* predicted risky driving (e.g., speeding). Although emotion regulation is not diagnostic for autism, emotion dysregulation is argued to underlie other diagnoses that often co-occur with autism, such as depression, anxiety, and attention deficit/hyperactivity disorder (ADHD), and can lead to functional impairment (Simonoff et al., 2008; Anderson et al., 2011; Mazefsky et al., 2013; Gotham et al., 2015). Thus, it is possible that emotion dysregulation would also be associated with driving difficulty (DD) in autistic drivers. Relatedly, a systematic review of driving factors for autism highlighted that novice autistic drivers experience unique challenges in driving confidence and should develop emotional coping mechanisms (Lindsay, 2017).

In addition to emotion dysregulation, negative affect itself may be related to DD. Previous studies have found that greater negative affect symptoms specific to anxious or depressed mood, for example, are associated with greater risky driving behaviors (Scott-Parker et al., 2011; Trógolo et al., 2014). Moreover, difficulty inhibiting strong negative emotions like anxiety or anger predicted risky driving behaviors (Ulleberg and Rundmo, 2003; Oltedal and Rundmo, 2006; Hayley et al., 2017). These results suggest that negative affect and emotion dysregulation may contribute to DD, although these studies have not been conducted in autistic samples so it is unknown how these factors are impacted in autism. Nonetheless, there is strong context for further study on the association of emotion dysregulation and negative affect with DD in autism, which could have implications for specialized supports when learning to drive.

In this study, we aimed to clarify relationships between emotion regulation, negative affect, and perceived DD in adolescents and young adults with and without autistic traits (AT). Specifically, this study tested the hypotheses that (1) perceived DD, emotion dysregulation, and negative affect scores would be greater in participants with AT and (2) there would be a positive correlation of perceived DD with emotion dysregulation and negative affect in the whole sample. Measures that evaluate these emotional processes may provide guidance and quantify potential challenges in novice drivers with AT.

## Method

### Participants

The study recruited individuals aged 15 years or older from the Virginia Tech (VT) Autism Clinic & Center for Autism Research registry and the community *via* e-mails and electronic fliers to participate in an online survey regarding driving attitudes from June 2015 to July 2017. Individuals who self-reported any diagnosis of autism comprised the AT group (*n* = 15), and those without any self-reported previous autism diagnosis comprised the NA group (*n* = 22). Participants were required to be able to read and understand the consent/assent forms and survey items. Parental consent and child assent were collected for child participants and informed consent from all adult participants. Informed consent was monitored, and all study procedures were approved by the VT Institutional Review Board. Participants were compensated up to $10.00 for completing online questionnaires. The total sample consisted of 37 individuals (*M* = 17.5 years, *SD* = 2.16, range: 15–24). A total of 65% identified as male and 81% as white (Table 1). The Autism Spectrum Quotient (AQ; Baron-Cohen et al., 2001) was further used to characterize the sample, with significantly higher autistic characteristics in the AT versus NA group (see section “The AQ” for more details).

**TABLE 1:** Descriptive variables.

	AT	NA	*t*/χ^2^
*N*	15	22	
Age (years), *M (SD)*	17.53 (1.85)	17.55 (2.42)	0.02
Sex			0.22
Male, *n*	10	14	
Female, *n*	4	8	
Other, *n*	1	0	
Race			0.40
White, *n*	14	16	
Black/African American, *n*	0	3	
Asian/Asian American, *n*	0	2	
Hispanic, *n*	0	1	
Other, *n*	1	0	
Highest completed education level			0.87
Currently HS, *n*	10	14	
Graduated HS, *n*	2	2	
Currently post-secondary, *n*	3	5	
Graduated graduate school, *n*	0	1	
Has a job	46.7%	40.9%	0.12
Hours per week, *M* (SD)	15.29 (9.69)	27.89 (12.77)	2.17*

*AT, autistic traits group; NA, non-autistic group; HS, high school.*

*p < 0.05.

Those in the AT group self-reported the following co-occurring conditions: ADHD (*n* = 7), anxiety disorder (*n* = 7), mild intellectual disability (*n* = 1), schizophrenia (*n* = 1), and obsessive-compulsive disorder (*n* = 1); those in the NA group only self-reported co-occurring ADHD (*n* = 3).

### Questionnaires

#### Driving Metrics

Participants provided driving metrics (e.g., enrollment of driver’s education, status and age of licensure), and rated perceived DD on a four-point scale (*1* = very easy to *4* = very difficult) of 15 situations they may encounter while driving: during the daytime, the nighttime, and dawn/dusk, in bad weather, heavy traffic, towns, cities/urban areas, construction zones, and familiar and unfamiliar areas, on a highway/interstate, with silent and talking passengers, without any passengers, and near pedestrians/bicyclists.

The perceived DD scale was developed for this study and presented scenarios predicted to potentially elicit higher perceived DD ratings, including driving at night, in bad weather, with passengers, near pedestrians, etc., along with scenarios such as driving in familiar areas or during the daytime that may elicit lower perceived DD ratings. Scenarios were selected based on findings from previous literature that reviewed driving scenarios that autistic people have reportedly struggled in Daly et al. (2014), Chee et al. (2017). Perceived DD items were analyzed individually across groups and averaged for a total score, where higher scores indicate greater perceived difficulty. Internal consistency for perceived DD for the current sample was acceptable for both groups (NA, Cronbach’s α = 0.88; AT, α = 0.76).

#### The AQ

The AQ is a 50-item, self-report questionnaire that rates autistic characteristics in adults as “definitely agree,” “slightly agree,” “slightly disagree,” or “definitely disagree” (Baron-Cohen et al., 2001). The AQ has reasonable construct validity. and was used to characterize autistic characteristics in this self-reported sample of participants (Baron-Cohen et al., 2001). In the current sample, the total score was significantly greater in the AT group (*M* = 31.13, *SD* = 7.16) than the NA group (*M* = 19.69, *SD* = 6.07), *t*(35) = 5.24, *p* < 0.001, *g* = 1.71, and internal consistency was acceptable for both groups (NA, α = 0.72; AT, α = 0.83).

#### DERS

The DERS is a 36-item, self-report questionnaire that assesses frequency of difficulties in emotion regulation, rated on a five-point Likert Scale (*1* = almost never to *5* = almost always) resulting in six dimensions of regulation: nonacceptance of emotional responses (*Nonacceptance*), difficulty engaging in goal-directed behavior (*Goals*), impulse control difficulties (*Impulse*), lack of emotional awareness (*Awareness*), limited access to emotion regulation strategies (*Strategies*), and lack of emotional clarity (*Clarity*; Gratz and Roemer, 2004). Some items were reverse coded, and responses were summed for a total score and six subscale scores. Higher scores indicate greater emotion regulation difficulty. The DERS demonstrated good psychometrics in two separate samples of college-aged adults, one autistic and one NA, (Gratz and Roemer, 2004; Conner and White, 2018). There was excellent internal consistency in our sample for both groups (NA, α = 0.90; AT, α = 0.94).

#### Depression, Anxiety, and Stress Scale

The Depression, Anxiety, and Stress Scale (DASS) is a 21-item, self-report questionnaire that measures the severity of negative affect consistent with depression, anxiety, and stress (Antony et al., 1998). Participants rated items pertaining to emotional states in the past week on a four-point scale (*0* = did not apply to me at all to *3* = applied to me very much/most of the time). Raw scores were summed and doubled creating a total score and three 7-item subscale scores (*Depression, Anxiety, Stress*). The *Depression* subscale measures hopelessness, lack of interest/involvement, and anhedonia. The *Anxiety* subscale measures autonomic arousal, bodily sensations, and subjective experience of anxious affect. The *Stress* subscale measures difficulty relaxing, being easily agitated, and irritability. The DASS demonstrated good psychometrics in autistic and NA adults recruited from clinical and community samples (Antony et al., 1998; Clara et al., 2001; Henry and Crawford, 2005; Nah et al., 2018). Our internal consistency was good for both groups (NA, α = 0.88; AT, α = 0.90).

### Statistical Plan

We performed analyses using SPSS (IBM SPSS Statistics for Windows, Version 26.0, 2019) with a two-tailed alpha = 0.05. We conducted independent-samples *t*-test and chi-square analyses to compare AT and NA groups in descriptive data, driving metrics, average total perceived DD score, and DERS and DASS total and subscale scores. To protect against Type-1 Error, we conducted Bonferroni corrections by setting the alpha at 0.003/0.008/0.017 for comparisons on the DD scale/DERS/DASS, respectively. For *t*-tests, we reported effect sizes as Hedge’s *g* to provide a magnitude of group differences. Effects equal to or greater than 0.2/0.5/0.8 were respectively considered small/medium/large (Cohen, 1988). We conducted Pearson correlations to quantify the relationship of perceived DD with the DERS and DASS in the full sample. Given that those with autism and co-occurring conditions may have more consequences on driving (e.g., Cox et al., 2016), we conducted additional one-way analyses of variance (ANOVA) with *post hoc* Bonferroni corrections that looked at AT and NA groups with and without anxiety and ADHD.

#### Sensitivity Power Analyses

We conducted sensitivity power analyses to indicate effect sizes that the current study was sufficiently powered to detect based on the current sample size and analytic approaches employed using G*Power 3.1 (Faul et al., 2007). For independent samples *t*-tests, the present study had sufficient statistical power (80%) to detect significant differences between the AT and NA groups that exceeded a large effect size (*g* ≥ 0.85). For chi-square analyses and Pearson correlations, respectively, the present study had a sufficient sample size to detect a significant χ^2^ difference of at least 3.84 and significant bivariate correlations of at least 0.40.

## Results

### Demographic Factors

There were no differences between the AT and NA groups on age, sex, or race (Table 1). Both groups reported similar levels of education, χ^2^(3, *N* = 37) = 0.87, *p* = 0.832, and having a job, χ^2^(1, *N* = 37) = 0.12, *p* = 0.729 (Table 1). Of those employed, the NA group worked significantly more hours per week, *t*(14) = 2.17, *p* = 0.048, *g* = 1.09.

### Driving Education and Behavior

Among the 30 participants (*n* = 13 AT; 17 NA) who were eligible for a driver’s license in Virginia (at least 16 years old), significantly fewer participants in the AT group reported having a current driver’s license than in the NA group (χ^2^ = 4.44, *p* = 0.035). Also, a significantly greater proportion of individuals in the AT group reported a delay in obtaining licensure than in the NA group (χ^2^ = 8.17, *p* = 0.004; Table 2). The most common reason for delay was intrinsically related (66.7%; e.g., fear/stress around driving) for the AT group compared to extrinsically related (50%; e.g., parental permission) for the NA group. The proportions of those with a current or past driver’s license or learner’s permit or without ever having a license or permit did not differ significantly between the AT and NA groups in the whole sample (χ^2^ = 4.46, *p* = 0.216; Table 2). Of those without a current driver’s license, AT participants expressed wanting a driver’s license (χ^2^ = 1.16, *p* = 0.283), and attended and completed driver’s education at the same rate as NA participants (χ^2^ = 2.34, *p* = 0.310; Table 2).

**TABLE 2:** Group differences on driving in participants 16 + years old.

	AT		NA	
	%	*n*	%	*n*
	Participants 16 + years old			
	*n* = 12^a^		*n* = 17	

Current driver’s license				
Yes	25.0	3	64.7	11
No	75.0	9	35.3	6
Delayed driver’s license				
Yes	69.2	9	17.6	3
No	30.8	4	82.4	14

	Whole sample				
	*n* = 14^b^		*n* = 22	

Current driver’s license	21.4	3	50.0	11
Current learner’s permit	35.7	5	31.8	7
Past driver’s license	7.1	1	–	0
Never had license or permit	35.7	5	18.2	4

Participants without a Current Driver’s License				
	*n* = 11		*n* = 11	

Want driver’s license^c^				
Yes	100.0	11	90.0	9
No	–	0	10.0	1
Driver’s education				
Completed	54.5	6	36.4	4
Currently attending	9.1	1	36.4	4
Has never attended	36.4	4	27.3	3

*AT, autistic traits group; NA, non-autistic group; %, percentage of AT/NA group.*

*^a^1 out of the 13 participants did not respond to these questions.*

*^b^1 out of the 15 AT participants did not respond to this question.*

*^c^1 NA participant did not answer this question.*

The average perceived DD score was not related to sex (*t*[34] = 0.22, *p* = 0.825), age (*t*[37] = 0.12, *p* = 0.509), or having a driver’s license (*t*[27] = 1.32, *p* = 0.199). On average, the AT group reported significantly greater perceived DD than the NA group (*t*[35] = −4.04, *p* < 0.001; Table 3). The AT group rated all 15 driving situations as more difficult than the NA group, and significantly more difficult for driving in towns and with talking passengers^2^ (Table 3).

**TABLE 3:** Group differences on DD, the DERS, and the DASS.

	AT	NA			*p*	effect size
	*M (SD)*	*M (SD)*	df	*t*		
DD overall average	2.45 (0.36)	1.89 (0.45)	35	–4.04	<0.001	1.35
Daytime	1.60 (0.50)	1.14 (0.35)	23^a^	–3.07	0.005	1.10
Nighttime	2.79 (0.69)	2.14 (0.77)	34	–2.54	0.016	0.87
Dawn or dusk	2.27 (0.45)	2.00 (0.81)	35	–1.15	0.260	0.38
Bad weather	3.13 (0.64)	2.86 (0.79)	34	–1.11	0.273	0.38
Heavy traffic	3.00 (0.75)	2.32 (0.99)	34	–2.37	0.024	0.75
Highway or interstate	2.47 (0.91)	1.85 (0.87)	33	–2.02	0.051	0.69
In towns	2.20 (0.67)	1.50 (0.51)	35	–3.59	0.001	1.20
In cities or urban areas	3.00 (0.75)	2.33 (0.96)	34	–2.22	0.033	0.75
In construction zones	2.87 (0.64)	2.14 (0.94)	35	–2.62	0.013	0.88
Familiar areas	1.27 (0.45)	1.14 (0.35)	35	–0.98	0.334	0.33
Unfamiliar areas	3.00 (0.84)	2.27 (0.70)	35	–2.85	0.007	0.95
Silent passengers	1.60 (0.63)	1.41 (0.66)	35	–0.87	0.388	0.29
Talking passengers	3.00 (0.84)	1.82 (0.79)	35	–4.33	<0.001	1.45
No passengers	1.67 (0.81)	1.15 (0.36)	18^b^	–2.28	0.035	0.86
Near pedestrians & bicyclists	2.87 (0.83)	2.32 (0.83)	35	–1.96	0.058	0.66
DERS Total score	99.67 (16.49)	89.05 (22.70)	35	–1.55	0.130	0.52
Nonacceptance	16.53 (7.59)	13.59 (6.34)	35	–1.24	0.209	0.43
Goals	15.60 (3.31)	14.09 (4.24)	35	–1.16	0.255	0.39
Impulse	17.00 (5.64)	10.50 (5.41)	35	–3.53	0.001	1.18
Awareness	17.87 (4.78)	20.14 (6.01)	35	1.22	0.230	0.41
Strategies	20.33 (5.23)	17.82 (6.43)	35	–1.26	0.217	0.42
Clarity	12.33 (3.09)	12.91 (3.19)	35	0.55	0.589	0.18
DASS Total score	34.30 (19.34)	18.73 (15.76)	35	–2.71	0.010	0.91
Depression	9.46 (7.23)	5.91 (8.27)	35	–1.35	0.186	0.45
Anxiety	8.67 (7.77)	5.55 (4.62)	35	–1.53	0.177	0.51
Stress	16.27 (9.47)	7.27 (6.28)	22^c^	–3.23	0.004	1.17

*AT, autistic traits group; NA, non-autistic group; DD, driving difficulty.*

*^a^For the “Daytime” item, Levene’s test indicated unequal variances so degrees of freedom were adjusted from 35 to 23 (F = 12.32, p = 0.001).*

*^b^For the “No Passengers” item, Levene’s test indicated unequal variances so degrees of freedom were adjusted from 33 to 18 (F = 7.99, p = 0.008).*

*^c^For the DASS Stress subscale, Levene’s test indicated unequal variances so degrees of freedom were adjusted from 35 to 22 (F = 7.19, p = 0.011).*

An exploratory ANOVA compared AT participants with and without a self-reported anxiety disorder to each other, and also to NA participants without an anxiety disorder on perceived DD. AT participants with and without co-occurring anxiety did not significantly differ from each other, but both AT groups rated significantly greater overall DD than the NA without anxiety group (Supplementary Table 1).

Similarly, an exploratory ANOVA compared AT participants with and without a self-reported ADHD to each other, and also to NA participants with and without ADHD on perceived DD. There were no significant differences between AT participants with and without co-occurring ADHD and between the NA participants with and without co-occurring ADHD. However, both AT groups indicated greater overall perceived DD than the NA without ADHD but not when compared to the NA with ADHD group (Supplementary Table 2).

### Emotion Regulation and Negative Affect

Independent sample *t*-tests showed significant differences between AT and NA groups on the DERS and DASS (Table 3). The AT group reported significantly higher scores on the DERS Impulse, DASS Total, and DASS Stress.

### Perceived Driving Difficulty and Emotionality

Across the whole sample, increased perceived DD was significantly (*r* = 0.52--0.53) correlated to increased DASS Total and Stress subscale^3^ (Table 4). The DERS subscales *Awareness*, *Strategies*, and *Clarity* were not significantly correlated with DD (Table 4).

**TABLE 4:** Correlation with perceived driving difficulty.

	Pearson correlation	*p*
DERS Total score	0.33	0.049
Nonacceptance	0.37	0.025
Goals	0.36	0.029
Impulse	0.40	0.014
Awareness	−0.21	0.212
Strategies	0.30	0.076
Clarity	−0.11	0.518
DASS Total score	0.53	0.001
Depression	0.37	0.025
Anxiety	0.38	0.021
Stress	0.52	<0.001

## Discussion

The current study examined the relationship of negative affect and emotion dysregulation with perceived DD in AT and NA participants who are interested in driving. The results of this study contribute to understanding the transition to adulthood for autistic individuals by highlighting the emotional difficulties they may face when achieving driving goals. These findings indicate significant group differences between individuals with and without autistic traits on driving metrics, perceived DD, emotion dysregulation, and negative affect.

Regarding their driving metrics, the AT group was less likely to obtain their driver’s license on time or at all which is consistent with previous studies of autistic individuals (Daly et al., 2014; Curry et al., 2018). Additionally, AT participants reported greater perceived DD and experienced significantly greater stress than NA individuals. A further look into the perceived DD scale showed that driving areas that AT participants rated significantly more difficult included scenarios that may be more demanding due to their unpredictable or distracting nature. These results support previous studies in autistic samples that have suggested that responding to unexpected situations is negatively impacted due to impaired emotionality (Sheppard et al., 2010; Cox et al., 2020). Exploratory analyses found that AT participants with a co-occurring disorder of anxiety or ADHD experienced overall DD when compared to the NA group; however, there were no differences within the AT groups regardless of co-occurring anxiety or ADHD, suggesting that perceived DD is not better explained by additional symptoms of inattention/hyperactivity and anxiety in AT participants, but could be the transdiagnostic features of emotion dysregulation and negative affect in autistic people. However, any endorsement of co-occurring conditions is self-reported which may not reflect the extent to which these symptoms could influence driving perceptions.

Correlations indicated that greater overall perceived DD was related to increased stress on the DASS. Although they were not significant after Bonferroni corrections, some of the DERS (Total and subscales: *Nonacceptance, Goals, Impulse*) were positively correlated with perceived DD. These DERS subscales may affect perceptions of demanding driving situations because their items map onto negative biases of emotional states (e.g., “When I am upset, I feel weak”—*Nonacceptance*), poor cognitive control (e.g., “I have difficulty concentrating”—*Goals*), and poor behavioral control (e.g., “I have difficulty controlling my behaviors”—Impulse), whereas the non-significant subscales target meta-cognitive concepts not directly related to views toward driving (e.g., “I believe that my feelings are important”—*Awareness*, “I believe there is nothing I can do to feel better”—*Strategies*, “I do not know how I am feeling”—*Clarity*). These findings are consistent with findings by Trógolo et al. (2014) that correlated risky driving styles to the *Nonacceptance, Goals*, and *Impulse* subscales and explained that broader internalizing symptomology (e.g., anxiety) can predict maladaptive driving styles, and further extends those findings to include autistic participants.

Moreover, of the current participants who reported a delay in obtaining their driver’s license, AT participants attributed their delay to negative internal feelings toward driving compared to the extrinsically related factors reported by those in the NA group. This related to the greater understanding that autistic individuals may experience a more intense reaction to negative affect (Mazefsky et al., 2013), and they converge with the results that showed an increase in negative affect and dysregulation from the DERS and DASS. Overall, these elevations begin to contribute to the relationship of emotion dysregulation and negative affect and perceived DD in new autistic drivers. These findings also align with the idea that autistic individuals may need specific support for socio-emotional differences when learning to drive as, for example, the intervention recently described in Baker-Ericzén et al. (2020). In addition, virtual reality driving simulations can transform generalized skill instruction for autistic learners, highlighted as a unique learning difficulty by Klin et al. (2003), by providing adaptive driving instructions that can respond to their real-time physiological arousal (Bian et al., 2019).

Lastly, the NA group had similar employment rates to the AT group; however, those in the NA group worked 12 more weekly hours on average than those in the AT group. Although our main objective was not to characterize employment, the current findings support the association between employment and independent driving considering that autistic drivers are generally provided more employment opportunities than their non-driving autistic peers (Feeley, 2010; Huang et al., 2012; Daly et al., 2014; Interagency Autism Coordinating Committee, 2017; Curry et al., 2018; Myers et al., 2019).

### Limitations

Due to the nature of this online study, we were unable to administer a gold-standard assessment battery [e.g., Autism Diagnostic Observation Schedule (Lord et al., 2012)] to confirm participants’ autism diagnostic status. Therefore, the primary limitation of this study was its use of a self-referred sample of autistic participants, and thus the sample may not be representative of the larger autism population. Although the average AQ total score for the AT group was right below the recommended cutoff (32) for autism, they scored significantly higher than the NA group which demonstrated group differences in autistic features between the two groups. Another limitation is the perceived DD scale that was developed for the purpose of this study, and therefore does not have established psychometrics. The current findings may overestimate driving behavior and attitudes due to selection bias such that those who are interested in or currently driving may have been more likely to participate. While the small sample size may present as a limitation, sensitivity power analyses demonstrated that the significant effects were large enough to be detected. Nonetheless, smaller effects may be overlooked and warrant further investigation with a larger sample.

Future directions include collecting more detailed data for a more complete profile of sensitive transition periods for autistic individuals. Future work would also benefit from conducting in-person visits with caregiver persepectives to allow for a comprehensive evaluation of cognitive, psychological, and objective driving abilities and confirm clinical group status. Conducting repeated visits would provide a prospective examination of how emotion regulation and negative affect relate to driving in autism. Finally, a larger sample would allow for more fine-grained analyses to examine the potential moderating role of associated mood and anxiety disorders in both AT and NA individuals as they prepare for driving. This study was limited by its cross-sectional descriptive design, and, therefore, a more comprehensive understanding of these relationships using longitudinal designs may contribute to developing interventions that address negative affect and emotion dysregulation related to driving.

## Conclusion

The current study is the first to examine the relationships of emotion regulation, negative affect, and perceived difficulties with driving in a sample of AT and NA participants and address its implications for adult functioning. AT participants reported greater overall perceived DD in different situations, and greater impairment in emotion dysregulation and negative affect compared to NA participants. These initial findings lend support to understanding emotion dysregulation and negative affect as important parts to learning to drive in general and may be especially relevant considering emotion processing differences in emerging autistic drivers. Independent driving is an age-appropriate skill for daily living for autistic adolescents and young adults and provides greater employment opportunities. Previous research has supported that daily living skills may be more predictive of later adult employment than other childhood factors (academic success, symptom severity, and IQ; Taylor and Mailick, 2014). Preparing autistic adolescents and adults for driving by addressing the impact of emotion regulation skills may be useful to consider promoting successful independent functioning and later adult outcomes.

## Data Availability Statement

The de-identified aggregate dataset may become available upon request. Requests to access the datasets should be directed to MF, mfok@vt.edu.

## Ethics Statement

The studies involving human participants were reviewed and approved by VT Institutional Review Board. Written informed consent to participate in this study was provided by the adult participants and the parents of child participants, and assent was provided by the child participants.

## Author Contributions

JO and AS conceptualized the study and oversaw data collection for analysis. MF contributed to the study conceptualization, data analysis, and interpretation of results. JO and TO contributed to the development and conceptualization of the perceived DD scale in the original study. All authors critically revised the article for intellectual content and approved the final version of the manuscript.

## Conflict of Interest

The authors declare that the research was conducted in the absence of any commercial or financial relationships that could be construed as a potential conflict of interest.

## Publisher’s Note

All claims expressed in this article are solely those of the authors and do not necessarily represent those of their affiliated organizations, or those of the publisher, the editors and the reviewers. Any product that may be evaluated in this article, or claim that may be made by its manufacturer, is not guaranteed or endorsed by the publisher.

## Footnotes

1 Autistic self-advocates recommend the use of identify-first (i.e., “autistic adults”) instead of person-first (i.e., “Adults with ASD”) language. Recent publications have recommended inclusive and non-ableist language such as using “autism” instead of “autism spectrum disorder” and “co-occurring” instead of “comorbid” (Botha et al., 2021; Bottema-Beutel et al., 2021; Bradshaw et al., 2021).

2 Before Bonferroni corrections, the AT group rated driving during the daytime, nighttime, heavy traffic, in cities/urban areas, construction zones, and unfamiliar areas, and without passengers as significantly higher.

3 When not corrected for multiple-comparison, all six DASS subscales, DERS Total, and DERS subscales for Nonacceptance, Goals, and Impulse were significantly positively correlated to perceived DD (*p*s = 0.014–0.049; Table 4).
