# Supplementary material for: Perceived Driving Difficulty, Negative Affect, and Emotion Dysregulation in Self-Identified Autistic Emerging Drivers
Source: Front Psychol. 2022 Jan 31;13:754776. doi: 10.3389/fpsyg.2022.754776 (PMC8841684; doi:10.3389/fpsyg.2022.754776)
Supplement: Supplementary file 1 [file Data_Sheet_1.docx]

**13 Supplementary Materials**

**13.1 Figure S1**

Bar graphs of significant group differences on DD


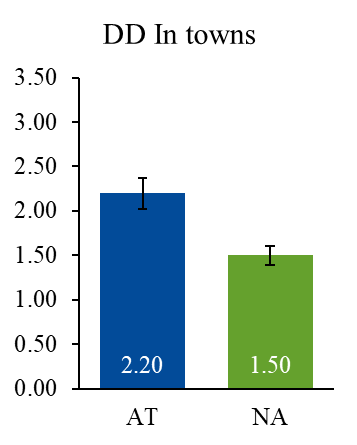

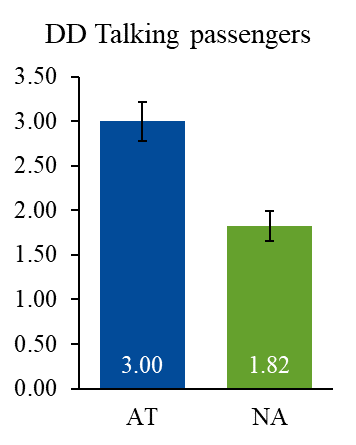


*Note*: DD=Driving Difficulty; AT=Autistic traits group; NA=Non-autistic group; bars indicate standard error

**13.2 Figure S2**

Bar graphs of significant group differences on the DERS Impulse and DASS Stress subscales and DASS Total Score


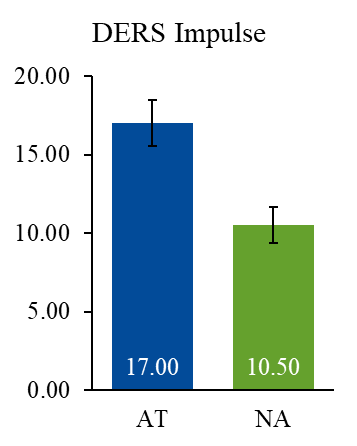


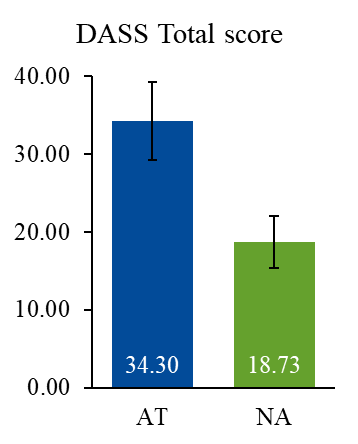

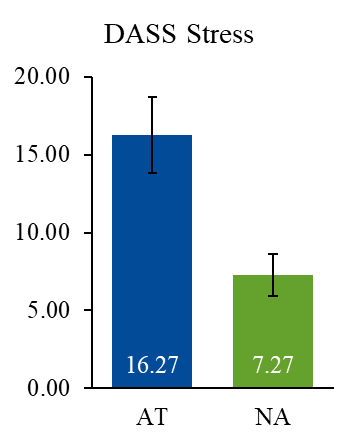


*Note*: AT=Autistic traits group; NA=Non-autistic group; bars indicate standard error

**13.3 Table S1**

| *Overall DD average between the AT with and without anxiety groups to the NA group.* | | | | | |  |
| --- | --- | --- | --- | --- | --- | --- |
|  |  |  |  |  |  |  |
|  | NA |  | AT | | |  |
|  | *n*=22 |  | with anxiety |  | without anxiety |  |
|  |  |  | *n*=7 |  | *n*=8 |  |
| *M* (SD) | 1.89 (0.10)^a,b^ |  | 2.41 (0.16)^a^ |  | 2.48 (0.11)^b^ |  |
| *Note*: DD= Driving Difficulty; superscript letters indicate significant group comparisons  ^a^*p*=.022; ^b^*p*=.005 | | | | | |  |
|  |  |  |  |  |  |  |

**13.4 Table S2**

| *Overall DD average between the AT with and without ADHD groups to the NA with ADHD and without ADHD groups.* | | | | | | | |
| --- | --- | --- | --- | --- | --- | --- | --- |
|  |  |  |  |  |  |  |  |
|  | NA | | |  | AT | | |
|  | with ADHD |  | without ADHD |  | with ADHD |  | without ADHD |
|  | *n*=3 |  | *n*=19 |  | *n*=7 |  | *n*=8 |
| *M* (SD) | 2.11 (0.71) |  | 1.85 (0.41)^a,b^ |  | 2.39 (0.36)^a^ |  | 2.50 (0.38)^b^ |
| *Note*: DD=Driving Difficulty; superscript letters indicate significant group comparisons  ^a^*p*=.040; ^b^*p*=.005 | | | | | | | |
|  |  |  |  |  |  |  |  |
